# Effectiveness of the World Health Organization Safe Childbirth Checklist (WHO-SCC) in preventing poor childbirth outcomes: a study protocol for a matched-pair cluster randomized control trial

**DOI:** 10.1186/s12889-021-11673-0

**Published:** 2021-09-10

**Authors:** Tieba Millogo, Kadidiatou Raïssa Kourouma, Aïssatou Diallo, Marie Laurette Agbre-Yace, Mamadou Diouldé Baldé, Seni Kouanda

**Affiliations:** 1 African Institute of Public Health (AIPH) & Institut de Recherche en Sciences de la Santé, Ouagadougou, Burkina Faso; 2 Institut National de Santé Publique (INSP) & Cellule de Recherche en Santé de la Reproduction, Abidjan, Côte d’Ivoire; 3 Cellule de Recherche en Santé de la Reproduction de Guinée (CERREGUI), Conakry, Guinea; 4 Institut de Recherche en Sciences de la Santé, Burkina Faso & African Institute of Public Health (AIPH), Ouagadougou, Burkina Faso

**Keywords:** WHO safe childbirth checklist, Quality of care, Burkina Faso, Côte d’Ivoire, Guinea

## Abstract

**Background:**

Women delivering in health facilities in sub-Saharan Africa and their newborns do not always receive proven interventions needed to prevent and/or adequately manage severe complications. The gaps in quality of care are increasingly pointed out as major contributing factor to the high and slow declining perinatal mortality rates. The World Health Organization Safe Childbirth Checklist (WHO-SCC), as a quality improvement strategy, targets low cost and easy to perform interventions and suits well with the context of limited resource settings. In this matched-pair cluster randomized controlled trial, we assess the effectiveness of the WHO-SCC in improving healthcare providers’ adherence to best practices and ultimately improving childbirth outcomes.

**Methods:**

This is a multi-country study. In each country we will carry out a matched-pair cluster randomized controlled trial whereby four pairs of regional hospitals will be randomized on a 1:1 basis to either the intervention or control group. A context specific WHO-SCC will be implemented in the intervention facilities along with trainings of healthcare providers on best childbirth practices and ongoing supportive supervisions. The standard of care will prevail in the control group. The primary outcome is a summary composite metric that combine the following poor childbirth outcomes: stillbirths, maternal deaths, early neonatal deaths, severe postpartum hemorrhage, maternal infections, early neonatal infections, prolonged obstructed labor, severe pre-eclampsia, uterine rupture in the health facility, eclampsia and maternal near miss. The occurrence of these outcomes will be ascertained in a sample of 2530 childbirth events in each country using data extraction. A secondary outcome of interest is the adherence of healthcare providers to evidence best practices. This will be measured through direct observations of a sample of 620 childbirth events in each country.

**Discussion:**

Our study has the potential to provide strong evidence on the effectiveness of the WHO-SCC, a low cost and easy to implement intervention that can be easily scaled up if found effective.

**Trial registration:**

The trial was registered in the Pan-African Clinical Trials Registry on 21st January 2020 under the following number: PACTR202001484669907. https://pactr.samrc.ac.za/TrialDisplay.aspx?TrialID=9662

## Background

The global efforts towards achievement of the 2015 Millennium Development Goals (MDGs) have led to substantial improvement in facility-based child births rates as compared to home deliveries, particularly in sub-Saharan Africa [1, 2], one of the most affected regions by maternal deaths in the world [3]. Yet, maternal and newborns mortality and morbidity rates remain unacceptably high and far above the expected levels [4]. Poor-quality of care in health facilities in low-resource settings is regarded as a major contributing factor to preventable maternal and newborn deaths and disability, justifying why the sustained increase in in-facility births has not been sufficient to reducing maternal and newborns deaths [5]. Quality of care improvement strategies appear to be important and needed as the health facility utilization is raising [6].

Checklist based strategies within the field of surgery have shown effectiveness in improving the adherence of healthcare providers to best practices and ultimately reducing post-surgery mortality and morbidity significantly and have now become a WHO-recommended best practice for all surgical facilities [7, 8]. The WHO Safe Childbirth Checklist (WHO-SCC) is a similar checklist initiative developed as a job aid tool that prompts/reminds healthcare providers on essential best practices at critical points during the course of childbirth care [9]. The checklist is organized around four key pause points during the course of childbirth and is intended to be a bedside tool for healthcare providers to improve the quality and safety of their care by adhering to proven and relatively inexpensive and easy to perform interventions. The rationale behind the checklist strategies in health care is that reminding healthcare providers of essential best practices at critical points can improve their delivery and ultimately help address the human factors of medical errors that arise from basing their implementation on the sole human memory [10]. Best practices are often neglected due to heavy workload or just due to routine or simply failure of human memory. Using a checklist systemizing best practices around the time of childbirth can help overcome these bottlenecks and hence promote quality care during childbirth and the immediate postpartum and neonatal period.

Previous studies have shown that healthcare providers significantly improve their delivery of proven effective and low-cost interventions during the time of childbirth and immediately after childbirth using the WHO-SCC [11–13].

**Fig. 1:**
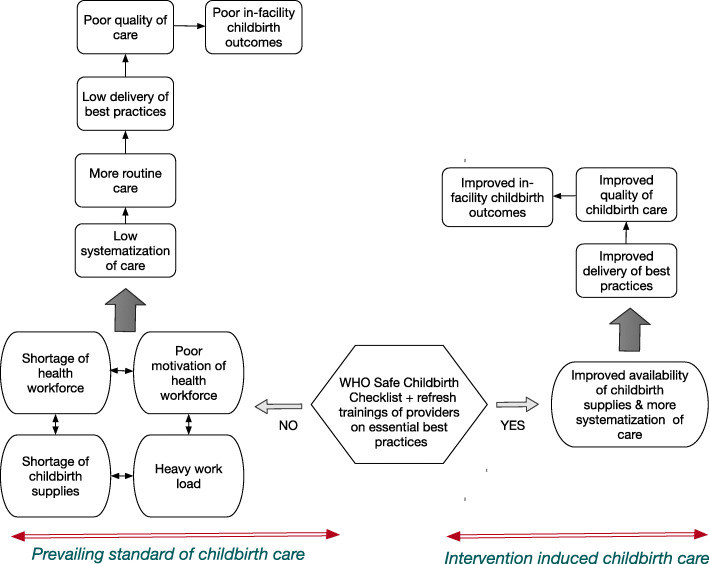
study conceptual framework. In this figure we depict the cascade leading to poor childbirth outcomes in the framework of the standard of childbirth care and how we postulate the use of the WHO-SCC will unravel this cascade leading to better childbirth outcomes

The WHO-SCC is a 29-items checklist which has had inputs from would-be users on the suitability of the content and the implementation strategy at the design phase [9]. The WHO-SCC was then tested in various contexts and has always shown, despite some inconsistencies in the magnitude of the effects [11, 14], effectiveness in improving healthcare providers’ adherence to essential best practices. All these studies emphasized the importance of contextual factors such as leadership commitment, staffing patterns of the health facility etc. and have encouraged context specific adaptation of the checklist (including content change and implementation strategy) in order to reflect local practice and foster ownership. The WHO-SCC targets easy to perform and low costs interventions, yet not always adhered to, as it was shown in a baseline assessment of healthcare providers ‘performance of essential best practices in Burkina Faso and Côte d’Ivoire [15]. Furthermore, the evidence on the effectiveness of the WHO SCC in ultimately improving childbirth outcomes is inconclusive [16] and a recent study suggests that the effects on childbirth outcomes would be more apparent at the referral level of the healthcare system [14]. Our study will seek to assess the effectiveness of a context-specific adapted WHO-SCC in improving healthcare providers’ adherence to EBPs and ultimately in improving childbirth outcomes in referral hospitals of the implementing countries. We postulate that there is an unfavorable cascade leading to poor childbirth outcomes that can be reversed using the WHO-SCC as shown in the study conceptual framework (see Fig. 1). This study was preceded by a first phase where we assessed the gaps in the quality of child birthing care and the acceptability and perceived usefulness of the WHO-SCC in Burkina Faso and Côte d’Ivoire. The results of the first phase showed important quality gaps in delivery care in both countries and the perceived usefulness and the feasibility of the WHO-SCC in both countries conditional on some modifications [15, 17].

### Study hypothesis and objectives

We hypothesized that the implementation of a country specific adapted WHO-SCC in referral hospitals, would increase the availability of safe childbirth supplies, the delivery of essential best childbirth practices to the mother-child pair around the time of childbirth, and ultimately leads to improved perinatal health outcomes.

The specific objectives of the study are:

- To measure the effectiveness of a modified WHO-SCC in improving healthcare providers’ adherence to EBPs during intrapartum and immediate postpartum care.
- To assess the effectiveness of modified WHO-SCC in improving the availability of safe childbirth supplies at health facilities levels.
- To assess the effectiveness of a modified WHO-SCC in preventing poor childbirth outcomes in under-resourced settings.

## Methods

### Trial design

The intervention will be delivered at health facility (cluster) level and outcomes measurements will be carried out at individual participant level. We will use a matched paired cluster randomized controlled trial design, where a pair of health facilities (one intervention and one control) are enrolled in the study at the same time. The study will be conducted in referral hospitals with gynaecologists and where surgical facilities for c-section and blood transfusion services are readily available. Eight (8) regional hospitals (4 intervention and 4 control sites) will be included in the study in each country, giving a total of 24 health facilities (12 in intervention and 12 in control arms) in the three countries. Within each matched pair of health facilities, one will be randomized to receive the intervention and one will receive the standard of care. Facilities will be matched using geographic location within the country, the size of the population covered by the health facility and the annual births volume of the facility. The study will combine two main components: a data extraction component (used to measure the incidence of poor childbirth outcomes) and a direct observation of childbirth practices (to assess the availability of safe childbirth supplies and the adherence of care providers to EBPs).

### Study settings

The study will be conducted in three west-African countries that carry on an important burden of poor childbirth outcomes: Burkina Faso, Guinea and Côte d’Ivoire. The first two are ranked as Low-Income Countries (LIC) per the World Bank ranking, while the latter is ranked as a Low Middle Income Country (LMIC). All these countries have made important progress in shifting deliveries from home to health facilities in recent years. However, these improvements in health facility utilization for childbirths were not followed by the expected improvement in childbirth outcomes. The Maternal Mortality Ratios (MMR) stall and remain unacceptably high and very far from all targets. The latest estimates of MMR were 320, 576 and 617 per 100,000 live births respectively for Burkina Faso, Guinea and Côte d’Ivoire. The neonatal mortality rates are also high for the three countries with respectively 28, 33 and 38 deaths per 1000 in Burkina Faso, Guinea and Côte d’Ivoire [18–20]. The general healthcare delivery system is dominated by the government owned health facilities and is organized in a similar way in each of the three countries, with at the top the national and teaching hospitals that serve as last referral level, at the middle the regional hospitals, and at the bottom the health district system that comprises several primary healthcare facilities organized around a district hospital. The private health sector is embryonic and mainly limited to large cities. The study will be conducted in regional hospitals.

In all three countries, a regional hospital is a last referral hospital for health facilities of various functional classification (primary healthcare facilities, district hospitals) in a particular administrative location called region. It is a level of care where enough capacities are available to adequately manage obstetric complications if they are timely detected as compared to primary healthcare facilities and district hospitals. Because the vast majority of women and their newborns timely detected with complications will not have to be referred to a higher level of care, exposing them to known bad conditions of transportation and related delays, the adoption of the SCC is expected to have more potential to impact morbidity and mortality at this level of care. There are 10 functioning regional hospitals in Burkina Faso, 17 in Côte d’Ivoire and 07 in Guinea. A medical center located in the capital city in Guinea (Conakry) and that has the same level of care as regional hospitals will selected to complement the study sites in Guinea.

### Intervention

The intervention will encompass: (i) the introduction of a modified WHO-SCC in health facilities, (ii) trainings of healthcare providers on good childbirth practices, (iii) and ongoing supportive supervisions. The modified WHO-SCC, as a job aid tool that prompts healthcare providers to remember EBPs that a woman and/or her new-born must receive at four critical junctures during intrapartum and immediate postpartum periods, will be adapted from the WHO-SCC to reflect local contexts and align to the national guidelines of each implementing country. Based on the results of the formative research that showed that the implementation of the WHO-SCC would be more effective if healthcare providers are trained on EBPs complemented by supportive supervisions, the modified WHO-SCC will be introduced in intervention health facilities following a coaching-based approach [12–14, 21]. The formative research also suggested that the content and the ordering of the items of the WHO-SCC should be revised to reflect local practice [17]. This adaptation process will be led by a Technical Advisory Group (TAG) that includes the research teams, the ministries of health and the associations of gynaecologists and paediatricians in each country. The content and wording of the items to be included in the WHO-SCC will be first reviewed and validated by the TAG in a one-day meeting. The resulting WHO-SCC will be pilot tested, and the results will be presented to the TAG in another one-day meeting for the finalization of the WHO-SCC to be implemented in each country. For the introduction of the WHO-SCC in health facilities we will use the coaching based approach with the change model that includes Engage, Launch and Support. Prior to the introduction of the checklist in each health facility, we will engage the administrative and medical leaders to discuss the current deficiencies in the delivery of EBPs to women and new-borns in the intrapartum and postpartum periods and the need for a quality improvement intervention. The results of the formative phase showed important quality-gaps in maternity care. The content of the training sessions will be informed by these quality-gaps and we will put more emphasis on areas needing more improvements. The head medical officer of the maternity ward along with the most experimented midwife in each intervention health facility will have a 2-day orientation training to the modified WHO-SCC and EBPs during childbirth. The trained midwife is meant to be the facility-based coach for day-to-day follow up of the utilization of the checklist after the launch. A one-day orientation session to the WHO-SCC will be organized on site for the staff members of each intervention facility and will be run by the head medical officer, the trained midwife with the support from the research team. After the introduction, the support will be assured by the facility-based coach who will identify barriers to the utilization of the checklist including issues with supplies and help overcome them on a day-to-day basis. New staff members appointed in the facility after the launch will automatically benefit from a 1-day orientation session leads by the head medical officer and the trained facility-based coach. The modified WHO-SCC will be introduced in intervention facilities using a combination of two approaches that were identified as the most effective and acceptable ways for its introduction during the formative research. The first way is to reproduce the modified WHO-SCC in a larger format that will be displayed in the delivery room at the bedside of the woman, allowing healthcare providers to have a glance on it at any time while caring for the mother-new-born pair. Similar approaches are used with the guidelines for the management of post-partum hemorrhage and were found effective by most of the healthcare providers during the formative phase. This approach, while effective in prompting on EBPs at any time, does not however, allow the utilization of the modified WHO-SCC as a documentation tool of EBPs in addition. To allow the utilization of the modified WHO-SCC as a documentation tool as well, we will combine the displaying in delivery room with the attachment of a copy to the partograph.

### Control group

The standard of care will prevail in the control health facilities. There is no childbirth checklist, or a similar tool being used at the moment in health facilities in all the three countries.

### Study participants and recruitment

Pregnant women admitted in the delivery room of participating health facilities for delivery labour during the study recruitment period will be included in the study along with their new-borns. Eligible pregnant women are those for whom a vaginal delivery was considered as first option at admission and the monitoring of the childbirth labour was started. The study will have two components: (i) the direct observation of childbirth practices and (ii) data extraction from patient management tools to assess childbirth outcomes.

- Direct observations of child birthing practices

The participants selection into the direct observation component of the study will follow the successive steps below:

- Screening
- Consenting eligible participants
- Pre-inclusion of eligible participants
- Final inclusion of participants after initial examination

Pregnant women presenting in the delivery room will be systematically screened by the data collectors for basic eligibility criteria. Pregnant women that meet the following criteria will not be selected for the consent process:

- Admission for a planned C-section
- Manifest advanced stage of the labor
- Minors (age < 18 years)

The data collectors will then consent and pre-include the consenting participants in the study at this stage. After the initial examination will be excluded from the study:

- Pregnant women for whom a decision of emergency reference is taken without administration of proper care

Eligible women at this stage are finally included in the study. Pre-inclusion data for women finally excluded will be discarded.

- Data extraction to assess childbirth outcomes

The data extraction component of the study will include data from all pregnant women that were admitted in the study period and for whom the labor was monitored in the health facility. We will exclude:

- All the women who were admitted in the child birthing room for elective caesarian section (before the labour has begun);
- All the women and their newborns that were transferred for additional care after a delivery outside of the participating health facility and those received for any other type of obstetric care not related to childbirth (abortions, miscarriages and antenatal care);
- Pregnant women referred to higher level of care upon admission without proper administration of care at the level of the participating health facility;
- Mother-newborn pairs for whom provider was prompted to adhere to best practices in order to prevent a life-threatening situation during the direct observation of childbirth practices.

The recruitment will be done by appointed data collectors in each health facility (direct observation) and the facility-based coaches (data extraction). The enrolment into the study for the direct observation of care practices will take place right at the admission in the health facility, before the initial examination is performed. The process of consenting participant will, however, not be caused to delay the health care and for participants that were not able to provide informed consent before initial examination we will not collect data for this pause-point. Data will be collected for the subsequent pause-points if we get informed consent from the women after initial examination. For the data extraction component of the study, the mother-newborn pairs will be consecutively recruited from 2 weeks after the launch of the intervention to 6 months or until the prespecified sample is reached for each health facility. For the direct observation of childbirth care practices, a sample of mother-new-born pairs will also be consecutively recruited among those admitted for childbirth labour in each health facility. This recruitment will start 2 months after the onset of the intervention in intervention facilities and will continue until the calculated sample size for each health facility is achieved.

### Study outcomes

#### Primary outcome

The primary outcome in this study is a summary composite metric of poor childbirth outcomes. The component outcomes of the summary outcome are those anticipated to be preventable by the correct utilization of the modified WHO-SCC. They will encompass in this study: stillbirths, maternal deaths, early neonatal deaths, severe postpartum hemorrhage, maternal infections, early neonatal infections, prolonged obstructed labor, severe pre-eclampsia, uterine rupture in the health facility, eclampsia and maternal near miss. The definitions for the above severe complications are based on WHO clinical and laboratory-based criteria [22].

The presence/absence of each of the above poor childbirth outcomes will be ascertained in each birth event using data extraction from registries and patients’ charts. Different data sources will be used to cross-validate the information. These include birth registries, partograph, temperature sheet and mothers’ health books.

Regarding all these items the standards in all countries are anticipated to be similar. Other secondary outcomes that will be analyzed separately include the use of transfusion services and the return within 7 days to the health facility after discharge for complications related to the mother or the new-born. Two waves of supplies ‘availability assessments (initial assessment prior to the launch of the intervention and final assessment at the end of the study) will be conducted in both intervention and control health facilities.

#### Secondary outcomes

The first secondary outcome is the level of adherence to EBPs by healthcare providers during childbirth and early postpartum. This will be measured as a rate of successful delivery of key EBPs included in the modified WHO-SCC at each birth event. The provision of key EBPs will be ascertained in a sample of birth events using direct observation with possible cross-validations with data from birth registries and mothers’ health books when necessary. The anticipated best practices to be assessed in this study include: the assessment for need of reference, the use of a partograph, assessment for history and actual bleeding, vision blurred, HIV status, correct use of magnesium sulfate, uterotonics and antibiotics, early initiation of breastfeeding and skin-to-skin contact, and discussions around family planning services.

The second secondary outcome is the rate of availability of essential safe childbirth supplies measured at health facility level. A facility assessment questionnaire will be used to collect data on the availability of essential safe childbirth supplies as baseline assessment prior to the onset of the study. Six months after the launch of the study an end line survey will be conducted using the same questionnaire.

### Sample size

Different sample size calculation will be carried out for different study outcomes.

#### The incidence of poor childbirth outcomes

The component outcomes of the summary primary outcome are relatively infrequent events. Looking at recent health system information data from the three countries, we estimated that at least one of the component outcomes would be observed in an average 15% of vaginal deliveries in the study settings. With a bilateral type I error set at 5%, and a half reduction in the incidence of the primary outcome for the intervention group considered sufficient to prove the effectiveness of the intervention, we would need to observe a minimum of 1200 childbirths in each group (4 clusters per group) for a statistical power of 80%, assuming an intra-class correlation coefficient of 0.01 [23] and an average cluster size of 300. The calculated sample size was adjusted for a non-response rate of 5%. The final required sample size per country is therefore of 2530 childbirth events, half in each trial arm.

#### The adherence to EBPs

Based on the results of a formative research that showed important variations (ranging from very low to very high) in the adherence to EBPs in Burkina Faso and Côte d’Ivoire (unpublished data), we assume an average and conservative adherence of 50%. Considering a type I error level of 5% and taking into account the study design, a sample size of 620 childbirth events would be necessary in each country to detect an effect size of 15 percentage point with a statistical power of 80%.

### Data collection and follow up procedures

Two data collection techniques will be used: a direct observation of healthcare providers’ performance of best practices and data extraction from various source documents to assess childbirth outcomes.

#### Direct observations

Data collectors with a medical background (nurses, midwives or medical students) will be recruited and trained by the research team in each country. The data collectors will not be staff members of the participating health facilities and will be solely appointed in the health facility for the purpose of the study. Medical students are those in the last year of their medical studies and that are awaiting the defense of their thesis. The trainings will last for 3 days in each country and include orientation to the WHO-SCC, mastering of the questionnaire through in-room role-plays and field testing. The data will be collected using a standardized questionnaire performed into electronic devices (tablets or smartphones). The questionnaire will capture in addition to the information on the items included in the modified WHO-SCC, the socio-demographic characteristics of the mothers and new-born along with that of health care providers. To achieve the calculated sample size, we anticipate the observation of 80 childbirth events in each health facility. Considering a minimum average of 2 eligible childbirth events per health facility and per 24 h, the observation will last for a maximum of 45 days in each health facility.

Two data collectors will be appointed permanently (day and night) in each regional hospital during the period of data collection to directly observe and document the healthcare providers’ practices. The observations for each birth event will take place at four critical junctures from admission to discharge: at the admission, before the woman starts pushing, soon after delivery and just before discharge from the hospital. The observation will be strictly passive with no interaction with the healthcare providers and the patients. However, the data collectors can alert the healthcare providers in the event of occurrence of a life-threatening complication. Furthermore, should healthcare providers not adhere to a standard putting mother-new-born pair at high risk (examples: no administration of ARTs, antibiotics, sulfate of magnesium when needed), the observer will intervene in a kindly manner to suggest that such practice may be worthwhile. The intervention of the observer will follow a suggestive process without any coercion on the healthcare provider being observed. In such a case it will be recommended to probe the healthcare provider questioning the relevance of such a practice. For example, what do you think of the administration of ARTs to this woman? Would that be relevant? The healthcare provider must not be driven to feel guilty or blamed for anything. Any suggestion deemed relevant will be made in an inconspicuous manner, meant to be a private communication between the observer and the relevant healthcare provider. If an EBP was adhered to only after a suggestion was made by the data collector, her/she will still record that particular EBP as not having been adhered to by the healthcare provider.

The observation will be conducted every day of the week and at any time without discontinuation until the target sample size is achieved. A data collector may observe only part of a birth event and the observation will be continued by another data collector. However, data on each critical juncture must be fully collected by the same data collector. Thus, once a data collector has initiated the observation for a given pause points, he/she can get off work only after having filled in all the items pertaining to that pause point.

#### Data extraction

The data extraction will be carried out using a standardized questionnaire performed into electronic devices. Using different sources of information including a modified birth registry, patients charts and maternal health books where available, we will collect socio-demographic and childbirth outcomes data in both control and intervention health facilities on all consecutive eligible women that were admitted for labour and delivery from 2 weeks after the launch of the intervention to 8 months. The data extraction in each health facility will be conducted twice-weekly by facility-based trained coaches. They will review data source documents to collect information on each eligible mother-new-born pair from admission to 7 days after delivery. Based on the calculated sample size, we will need to include and extract data on 320 childbirth events in each health facility. Based on the minimum average of two childbirths events per 24 h, each health facility is expected to have cared for at least 320 pregnant women in 6 months from the launch of the study.

### Statistical methods

The analysis will be per intention to treat. Descriptive statistics for the study sample will be computed using means and standard deviations or medians and interquartile ranges (IQR) for continuous covariates, and proportions will be used to describe categorical variables. We will make use of the Rao-Scott chi-square test statistic, which adjusts for the matched-pair, cluster randomization scheme [24], to compare the rates of the primary and secondary outcomes in the comparison and intervention groups if the randomization scheme was found effective. Possible multivariable modeling techniques to correct for potential imbalances between study groups include Generalized estimating equations, multilevel Poisson modeling and multilevel cox models. The data analysis will be carried out using Stata 15.1 software. A *p*-value< 0.05 will be necessary to consider intervention and comparison groups significantly different.

### Quality control

A comprehensive quality assurance and quality control mechanism will be put in place to ensure that the best possible data is collected and entered into the database. To ensure that the data collection is carried out in the same ways, using the same criteria and standards for measurements, all the data collectors will be trained together, and standardized definitions of cases will be provided. A data collector manual will be elaborated with detailed description of the standardized operating procedures (SOPs) to serve as a day-to-day guide for the data collection. A close supervision mechanism of the data collection process will be put in place, whereby data collectors are regularly visited by the research team and a sample of completed cases (5%) will be double-checked once a week for completeness, accuracy and consistency. Quality check controls will be included in the data entry program using CSPro to exclude outliers. Inconsistent data will be returned to the field for correction before validation.

### Dissemination plans

We see our study as part of the global efforts to construct the body of evidence on the acceptability, feasibility and effectiveness of the modified WHO-SCC.

At the local level the findings will be shared during a one-day workshop with all the heads of participating health facilities and the heads of the maternity units. In turn the heads of health facilities and maternity units will share the results with their respective staff. The findings will be disseminated at the larger level of the country through a dissemination workshop that will be convened in collaboration with the Ministry of Health to involve all the relevant stakeholders and help prepare the scaling up if the findings are conclusive. In the event, the modified WHO-SCC is found effective, steps will be taken also in the different countries as to the integration of the modified WHO-SCC into training curriculum for nurses and midwives and in medical schools. The MOH in Burkina Faso has set up a knowledge transfer unit to create a breach between researchers and policy makers and implementers. The unit advises the MOH on evidence-based practices. We intend also to seize the opportunity of the existence of this unit and potential similar bodies in the other countries, to help with the advocacy towards adoption and scaling up of the modified WHO-SCC in the different countries. The dissemination to the wider scientific community will be done through scientific publications and communications during international conferences.

### Plans for communicating important protocol amendments to relevant parties

Should important modifications to the study protocol deemed necessary, they will be discussed and agreed upon by all the study team members in the three countries. Substantive modifications include without limitation, changes to the study objectives, the eligibility criteria, the sample sizes, the outcomes, or the statistical analyses. Any such changes will be documented and published as amendments to the current protocol. Amendments shall be made also to the trial registry entry in PACTR.

## Discussion

Poor quality of care during institutional births in low resource settings is a main contributing factor towards preventable childbirth-related poor outcomes. Accelerating progress in mother and child health is one of the great expectations of the new SDG era [25].

Building upon the success of checklist strategies in the field of surgery [7, 8], the WHO-SCC aims to address major causes of maternal mortality through improved delivery of known effective, low cost and easy to implement interventions [9]. As a randomized controlled trial Our study has the potential to provide strong evidence on its effectiveness and we have accounted for the limitations of previous similar studies in deciding to conduct this study in referral hospitals. An important weakness of this study design is the external validity. We believe, however, that our study will have an improved external validity because of its multi-country nature allowing the assessment different contexts. The trial will be conducted in the context of the current COVID-19 pandemic that might affect the study in different ways: travels restrictions, modifications in health facilities utilization etc. However, we can reasonably assume that it will have comparable effects on both intervention and control groups in each country. A great advantage of the WHO-SCC is the low cost of implementation. If found effective, the scaling up will not incur unaffordable cost for the Ministries of Health of implementing countries.

### Trial status

This is the protocol version 1 submitted in February 2021. The trial was approved by the ethics review committees of the implementing countries and lastly by that of WHO. We are starting the planning and pilot phases. The recruitment is anticipated to start in early April and will be completed by November 2021.

## Data Availability

Not applicable.

## Abbreviations

WHO-SCC: World Health Organization Safe Childbirth Checklist
SDG: Sustainable development goals
MDGs: Millennium development goals
